# Dose-dependent effects of siRNA-mediated inhibition of SCAP on PCSK9, LDLR, and plasma lipids in mouse and rhesus monkey[S]

**DOI:** 10.1194/jlr.M071498

**Published:** 2016-11-28

**Authors:** Kristian K. Jensen, Marija Tadin-Strapps, Sheng-ping Wang, James Hubert, Yanqing Kan, Yong Ma, David G. McLaren, Stephen F. Previs, Kithsiri B. Herath, Ablatt Mahsut, Andy Liaw, Shubing Wang, Steven J. Stout, CarolAnn Keohan, Gail Forrest, David Coelho, Satya Yendluri, Stephanie Williams, Martin Koser, Steven Bartz, Karen O. Akinsanya, Shirly Pinto

**Affiliations:** Cardiometabolic Disease* Merck & Co. Inc., Kenilworth, NJ; Pharmacology,** Merck & Co. Inc., Kenilworth, NJ; Genetics and Pharmacogenomics,† Merck & Co. Inc., Boston, MA; Sirna Therapeutics§ Merck & Co. Inc., San Francisco, CA; Business Development and Licensing,*** Merck & Co. Inc., San Francisco, CA; Biostatistics,†† Merck & Co. Inc., Rahway, NJ; RNA Therapeutics,§§ Merck & Co. Inc., West Point, PA

**Keywords:** lipid and lipoprotein metabolism, metabolic disease, lipids/liver, animal models, low density lipoprotein, drug therapy, cholesterol, SREBP cleavage-activating protein, cardiometabolic disease, dyslipidemia, small interfering ribonucleic acid, proprotein convertase subtilisin kexin type 9

## Abstract

SREBP cleavage-activating protein (SCAP) is a key protein in the regulation of lipid metabolism and a potential target for treatment of dyslipidemia. SCAP is required for activation of the transcription factors SREBP-1 and -2. SREBPs regulate the expression of genes involved in fatty acid and cholesterol biosynthesis, and LDL-C clearance through the regulation of LDL receptor (LDLR) and PCSK9 expression. To further test the potential of SCAP as a novel target for treatment of dyslipidemia, we used siRNAs to inhibit hepatic SCAP expression and assess the effect on PCSK9, LDLR, and lipids in mice and rhesus monkeys. In mice, robust liver Scap mRNA knockdown (KD) was achieved, accompanied by dose-dependent reduction in SREBP-regulated gene expression, de novo lipogenesis, and plasma PCSK9 and lipids. In rhesus monkeys, over 90% SCAP mRNA KD was achieved resulting in approximately 75, 50, and 50% reduction of plasma PCSK9, TG, and LDL-C, respectively. Inhibition of SCAP function was demonstrated by reduced expression of SREBP-regulated genes and de novo lipogenesis. In conclusion, siRNA-mediated inhibition of SCAP resulted in a significant reduction in circulating PCSK9 and LDL-C in rodent and primate models supporting SCAP as a novel target for the treatment of dyslipidemia.

SREBP cleavage-activating protein (SCAP) is a well-characterized key regulator of lipid metabolism (1, 2). Its effects on lipid metabolism are mediated through interaction with the SREBP transcription factors, which activate the expression of genes involved in fatty acid, TG, and cholesterol biosynthesis, as well as clearance of lipoproteins through regulation of LDL receptor (LDLR) and PCSK9 gene expression (3, 4). In the liver, SCAP integrates cholesterol sensing as well as insulin signaling into the regulation of SREBP transcriptional activity (5, 6).

Deletion of liver Scap in mouse models leads to a reduction in the expression of SREBP-regulated genes, de novo free fatty acid and cholesterol synthesis, liver TG levels and plasma lipid levels (7, 8). Overexpression of SREBP-1c in mice results in fatty liver, increased VLDL secretion, and increased plasma lipid levels (9, 10). Further, in rodent models of type 2 diabetes mellitus, augmented insulin signaling causes an induction of SREBP-1c activation, fatty acid synthesis, and accumulation of TG in the liver, as well as increased VLDL secretion (5, 11). In hamsters fed a high-sucrose diet, Scap knockdown (KD) by siRNA reduced the expression of SREBP-regulated genes, normalized liver and plasma TG levels, and reduced TG secretion. Ldlr and Pcsk9 mRNA levels were both reduced by Scap mRNA KD; however, LDLR protein levels were maintained, probably due to reduced levels of PCSK9 in circulation (8). Based on these observations from preclinical models, targeting SCAP may be of therapeutic benefit for treatment of dyslipidemia and fatty liver disease.

Because of the complex role of SCAP in regulation of lipid metabolism, it is important to demonstrate the translatability of the rodent findings into a higher species with lipid metabolism pathways more closely resembling that of humans. Specifically, because inhibition of SCAP negatively regulates both LDLR and PCSK9 mRNA expression, and PCSK9 is a negative regulator of LDLR, it is critical to determine the effect of targeting SCAP on the balance of LDLR and PCSK9 and how this ultimately affects LDL-C levels in such a model. Rhesus monkeys have been extensively used as a model for human lipid metabolism (12–14). Here, we describe the effects of hepatic SCAP KD on SREBP activity and lipid metabolism in rhesus monkeys using siRNA targeting. In parallel, we examined the effects of SCAP KD on SREBP pathway activity and lipid metabolism in mice. Our studies investigate the dose-dependent effects of hepatic SCAP inhibition on the balance of PCSK9 and LDLR expression. We demonstrate that SCAP siRNA-mediated KD results in reduction in plasma PCSK9, TG, and LDL-C in mice and rhesus monkeys.

## MATERIALS AND METHODS

### siRNA synthesis, lead selection, and lipid nanoparticle preparation

For mouse siRNA identification, 42 sequences were designed against mouse Scap transcript NM_001103162. For nonhuman primate (NHP) siRNA identification, 84 sequences were designed to have 100% human/rhesus homology using human SCAP transcript NM_012235 and rhesus SCAP transcript XM_001100342. Oligos were synthesized and screened in mouse Hela 1-6 cells (ATCC, Manassas, VA) and rhesus LLC-MK2 cells (ATCC) as previously described (15). The sequence of mouse and NHP lead SCAP siRNAs are shown in **Table 1** (all in 5′-3′ direction). siRNA oligos contained the following chemical modification added to the 2’ position of the ribose sugar where indicated: deoxy (d), 2’ fluoro (flu), or 2’ O-methyl (ome). Modification abbreviations are given immediately preceding the base to which they were applied. Passenger strands are blocked with an inverted abasic nucleotide on the 5′ and 3′ ends (iB). The nontargeting (nt) control siRNAs (nt controls) used in the experiments are listed in **Table 2** (all in 5′-3′ direction). PCSK9 siRNA oligo used in NHP studies and Pcsk9 and ApoB oligo used in mouse studies were described previously (15, 16).

**TABLE 1.** List of lead SCAP siRNA oligos used in mouse and NHP studies

siRNA	Guide Strand	Passenger Strand
Mouse Scap (m-Scap-1)	rU;rG;rU;fluC;omeG;omeA;fluU;fluU;omeA;omeA;omeG;fluC;omeA;rG;omeG;fluU;omeG;omeA;omeG;omeU;omeU	iB;fluC;fluU;fluC;dA;fluC;fluC;fluU;dG;fluC;fluU;fluU;dA;dA;fluU;fluC;dG;dA;fluC;dA;dT;dT;iB
NHP SCAP-1	rG;rU;rU;fluG;fluG;rU;fluG;rU;rC;fluA;fluA;rU;rU;fluA;fluA;fluG;rC;fluA;fluG;omeUs;omeU	iB;fluC;fluU;fluG;omeC;rU;rU;fluA;fluA;omeU;rU;fluG;fluA;rC;fluA;omeC;omeC;fluA;fluA;fluC;omeUs;omeU;iB
NHP SCAP-2	rU;rA;rU;fluA;rC;rC;fluA;fluG;fluG;fluA;rU;fluG;rC;rC;fluA;fluA;rU;rC;rC;omeUs;omeU	iB;fluG;fluG;fluA;fluU;rU;omeG;fluG;omeC;fluA;omeU;omeC;omeC;fluU;fluG;omeG;rU;rA;fluU;fluA;omeUs;omeU;iB

**TABLE 2.** The nt control siRNAs used in mouse and NHP studies

siRNA	Guide Strand	Passenger Strand
nt control-1	rU;rC;rG;omeA;fluC;fluC;omeG;omeA;fluU;omeA;fluU;omeA;omeA;omeG;omeG;fluC;omeG;omeA;fluC;omeU;omeU	iB;dG;fluU;fluC;dG;fluC;fluC;fluU;fluU;dA;fluU;dA;fluU;fluC;dG;dG;fluU;fluC;dG;dA;dT;dT;iB
nt control-2	rU;rA;rU;fluC;omeG;omeA;fluC;omeG;fluU;omeG;fluU;fluC;fluC;omeA;omeG;fluC;fluU;omeA;omeG;omeU;omeU	iB;fluC;fluU;dA;dG;fluC;fluU;dG;dG;dA;fluC;dA;fluC;dG;fluU;fluC;dG;dA;fluU;dA;dT;dT;iB

For in vivo studies, lead siRNA oligos were formulated in lipid nanoparticles (LNP) as previously reported (15, 17). Briefly, siRNAs were incorporated into the LNPs with high encapsulation efficiency by mixing siRNA in citrate buffer with an ethanolic solution of the lipid mixture, followed by a stepwise diafiltration process. The siRNA concentration in LNPs was determined by a gradient strong anion-exchange method. Encapsulation efficiency of siRNA was greater than 90% for all LNP formulations, as measured by the SYBR gold fluorimetric method. All siRNA LNP formulations were delivered systemically using an intravenous route.

### In vivo siRNA characterization in C57BL/6 mice

All procedures described below were approved by the Merck Research Laboratories Institutional Animal Care and Use Committee and carried out in accordance with the Guide for the Care and Use of Laboratory Animals. Male C57BL/6 mice were obtained from Taconic Farms (Germantown, NY). Mice were maintained in a 12/12 h light-dark cycle with free access to food and water in group housing conditions in a temperature-controlled environment (22°C). All mice were maintained on regular rodent chow (7012; Teklad, Madison, WI; 5% dietary fat; 3.75 kcal/g).

C57BL/6 mice (n = 4) received a single intravenous dose of 1 mg/kg of Scap siRNA-LNP, nt siRNA-LNP (nt control-1), or vehicle control. Animals were dosed via tail vein injections. On days 7, 14, 21, and 28 after siRNA dosing, the mice were euthanized with CO_2_ and liver samples were collected. Scap mRNA levels were measured as previously described (18). For the siRNA dose titration study, male C57BL/6 mice (n = 8) were dosed intravenously on day 0 with vehicle control, nt siRNA (0.5 mg/kg), or different doses of Scap siRNA (0.03125, 0.0625, 0.125, and 0.5 mg/kg). On day 10, food was removed in the morning for 4 h following which mice were dosed with deuterated water (D_2_O) (Sigma, St. Louis, MO) at 20 ml/kg intraperitoneally. Four hours post D_2_O injection, mice were euthanized with CO_2_ and EDTA plasma was collected via cardiac puncture and was reserved for lipids, circulating PCSK9, and fatty acid/cholesterol synthesis analyses. Livers were collected either in RNAlater or snap-frozen in liquid nitrogen and stored at −80°C for gene expression/protein analysis.

TaqMan quantitative (q)PCR was done on an ABI 7900 real-time PCR system using commercially available TaqMan probes and primers (Applied Biosystems mouse GAPDH, catalog #4352339E; mouse Scap, assay Mm01250176_m1; mouse Pcsk9, assay Mm00463738_m1; mouse Ldlr, assay Mm01177349_m1). Data analysis was performed as described previously (15).

### Hepatic TG secretion in C57BL/6 mice

On day 0, mice were dosed via tail vein injection with either a control siRNA at 0.5 mg/kg, or Scap siRNA at 0.0625, 0.125, and 0.5 mg/kg. One group of mice was dosed with ApoB siRNA at 1 mg/kg (iv). On day 10, food was removed in the morning for 4 h following which all mice were administered an intravenous dose of 50 mg/kg (10 ml/kg) of [^13^C_18_]oleic acid (Cambridge Isotope Labs) in 8% fatty acid-free BSA. Serial blood samples were obtained via tail nick at *t* = 0 (pre-tracer) and 15, 30, 45, 60, and 90 min post-tracer administration. The concentrations of the M_0_, M_18_, and M_36_ isotopologues of TG 52:2 were determined by LC-MS/MS as previously described (19). In order to determine the synthesis rate of newly formed TG 52:2, data at each time point from *t* = 15 min to *t* = 90 min were plotted as enrichments; the decay in enrichment (reflective of the rate of new TG synthesis) was fit to a mono-exponential decay function to determine a value for the fractional synthetic rate (k). The rate of production was then determined as multiplied by the average steady-state concentration of TG 52:2 from 15 to 90 min (20).

### Simvastatin and Scap siRNA cotreatment study

Transgenic C57BL/6 mice carrying the human CETP gene, including the natural flanking region (NFR) of the human CETP gene (NFR-CETP) (21), were obtained from Taconic Farms (Germantown, NY). NFR-CETP mice were dosed orally daily with 100 mg/kg simvastatin (Sigma; S6196) or vehicle (0.5% methylcellulose) starting on day 0. On day 4, mice were dosed via tail vein injection with control siRNA at 0.5 mg/kg, Pcsk9 siRNA at 1 mg/kg, or Scap siRNA at 0.0625, 0.125, and 0.5 mg/kg. On day 14, food was removed in the morning for 4 h. Mice were then euthanized with CO_2_ and EDTA plasma was collected via cardiac puncture and livers were collected either in RNAlater or snap-frozen in liquid nitrogen and stored at −80°C for gene expression/protein analysis.

### Plasma lipids, LDLR Western blot, and PCSK9 measurements in mice

Plasma total cholesterol (total cholesterol E; Wako Diagnostics) and TG (Infinity; Thermo) were measured by standard biochemical methods using commercially available enzymatic colorimetric kits according to the supplied product’s protocol. Lipoproteins were fractionated by FPLC (22). The column effluent was mixed with total cholesterol E enzymatic reagent (Wako Diagnostics), and absorbance at 600 nm was continuously recorded. The first, second, and third peak were attributed to VLDL, LDL, and HDL, respectively. Lipoprotein cholesterol levels were calculated as percent AUC of each peak × total cholesterol.

For LDLR Western blot, liver samples were homogenized on a MP Fast Prep-24 tissue homogenizer in 1,000 ul T-PER (Fisher catalog number 78510) with 1× Halt protease inhibitor (Fisher catalog number 78425) using 2 ml tubes (MP Biomedicals; Fast Prep-24 Lysing matrix D, 116913-500). Protein concentration was measured by BCA method. Liver lysate protein (100 ug) was separated on NuPAGE 4–12% Bis-Tris Gel (Invitrogen NP0322) and transferred using iBLOT (Invitrogen). Membranes were blocked at room temperature for 1–2 h using Odyssey infrared imaging system blocking buffer (part #927-40000) and incubated with Ldlr and β-actin antibodies [rabbit mAb to LDLr (EP1553Y), Abcam 52818; mouse mAb β-actin, Sigma A1978) at 4°C overnight, and then with secondary antibodies [donkey anti-rabbit IRDye 680 (Li-Cor catalog #926-32223) and donkey anti-mouse IRDye 800cw (Li-Cor, Cat# 926-32212)] at room temperature for 1 h. Band intensities were quantified on an Odyssey infrared imaging system (Li-Cor).

PCSK9 was measured using an in-house-developed ELISA assay (12). Briefly, the monoclonal antibodies, H23 and B20, were used as coating antibody (at 5 ug/ml) and detection antibody (1 ug/ml of the biotinylated form), respectively, for mouse PCSK9. Similarly, monoclonal antibody E07 was used as capturing antibody for NHP PCSK9 with B20 as detection antibody. Samples were assayed at 1:8 dilution and purified mouse PCSK9 protein was utilized for making standard curves. The ELISA assay was carried out in the standard procedure for the DELFIA detection system (Perkin Elmer), with the plates read on a Perkin Elmer EnVision 2103 multi-label reader.

### In vivo siRNA characterization in rhesus macaque monkeys

All NHP studies were conducted at New Iberia Research Center (NIRC) with the approval by NIRC’s and Merck’s Institutional Animal Care and Use Committees. The NIRC facility is accredited by the Association for Assessment and Accreditation of Laboratory Animal Care. Sexually mature male and female lean rhesus macaque monkeys weighing approximately 6–8 kg at the time of the study were randomized into groups based on prestudy body weight and serum lipid levels. Three rhesus experiments were performed: *1*) siRNA sequence selection study; *2*) siRNA KD duration study; and *3*) siRNA dose-titration study. For all three studies, animals were dosed intravenously based on body surface area with a single-rate controlled timed 2 min infusion. Blood samples were taken as alert procedure and percutaneous liver biopsies as sedated procedure. In each study, serum analysis for clinical chemistry, hematology, and coagulation parameters was performed for each blood collection time point, and the level of hepatic SCAP expression at each biopsy time point.

For the sequence selection study, animals received a 26.7 mg/m^2^ (approximately 2 mg/kg equivalent) dose of LNP-encapsulated siRNAs (n = 4 per group). Blood samples were taken on days −4 and 0 (prior to dosing) and on days 3, 5, 6, 7, 9, 14, 19, 21, and 28 following LNP injections. Percutaneous liver biopsies were performed on days −4 prior, 7, 14, and 28. Total cholesterol, LDL-C, and HDL-C were all measured directly from plasma using the Siemens Dimension assays for Clinical Chemistry Analyzer: CHOL, ALDL, and AHDL, respectively (Siemens, Princeton, NJ). Plasma PCSK9 levels were measured as described (12). TaqMan qPCR was done on an ABI 7900 real-time PCR system using the following primer pairs commercially obtained from ABI: Rh02621745_g1 (GAPDH), Rh02863945_m1 (SCAP), Rh03418189_m1 (PCSK9), Rh02828936_m1 (LDLR). Target KD in the liver was determined as previously described (17).

For the follow up siRNA KD duration study, group size was increased to 10 per group for SCAP (NHP SCAP-1) and nt control (nt control-2) siRNA-LNP. PCSK9 siRNA was used as a positive control in the study (n = 4). Animals received a 26.7 mg/m^2^ dose of LNP-encapsulated siRNAs. Blood samples were taken on days −7 and 0 (prior to dosing), 3, 7, 10, 14, 16, 17, 18, 21, 25, 28, 35, and 48 following LNP injection. In order to assess the effect of hepatic SCAP KD on palmitate and cholesterol synthesis, animals were administered D_2_O (4 ml/kg D_2_O by oral gavage) on day 16. Blood samples for the determination of palmitate and cholesterol synthesis were collected as fasted bleeds at −0.5 h and +4 h, +24 h, +48 h, and +5 days relative to D_2_O dosing. Percutaneous liver biopsies were performed on days −7, 7, 14, and 28. Serum analysis and liver TaqMan RT-PCR were done as described above.

The siRNA dose titration study was done using three dose levels of SCAP siRNA oligo: 26.7 mg/m^2^ (n = 4), 13.35 mg/m^2^ (n = 8), and 6.68 mg/m^2^ (n = 8). The dose levels were determined based on body surface area and approximately corresponded to 2, 1, and 0.5 mg/kg, respectively. The nt control-2 was dosed at 13.35 mg/m^2^ (n = 8). Blood samples were taken on days −2 and 0 (prior to dosing) and days 2, 3, 7, 8, 9, 10, 12, 14, 16, 17, 18, 19, 21, 23, and 26 following LNP injection. Percutaneous liver biopsy procedures were performed on day −2 (prior) and days 3, 12, and 26 post dosing. Serum analysis and liver TaqMan RT-PCR were done as described above.

### Determination of plasma palmitate and cholesterol synthesis

Deuterium enrichment in plasma water (precursor labeling) was determined by GC-MS (23) and enrichment in plasma palmitate and cholesterol (product labeling) was determined either by GC-MS for mouse samples (24) or GC-pyrolysis-isotope ratio mass spectrometry for rhesus samples (GC-p-IRMS) (25). Data analysis for the quantification of lipid synthesis was as previously described (24).

### Analysis of gene expression in rhesus liver samples

A 384-well format custom-designed rhesus PCR array was developed in collaboration with SABiosciences-Qiagen. Genes representing the major lipid and glucose metabolism pathways as well as genes involved in apoptosis, autophagy, and inflammatory pathways were selected based on publicly available canonical pathway databases (KEGG, GeneGO, and Ingenuity). A total of 372 individual genes and five housekeeping genes were assayed. Rhesus Genomic DNA Contamination primer control, reverse transcription control (duplicate), and positive PCR control (duplicate) were included on the array as part of the SABiosciences-Qiagen standard array setup. The array is available from SABiosciences-Qiagen, catalog number CAPQ09560. Real-time PCR was performed as previously described (24). ACTB, PPIA, GUSB, GAPDH, and RPL13A were used as housekeeping control genes.

### Statistical analysis

**Fig. 1.**
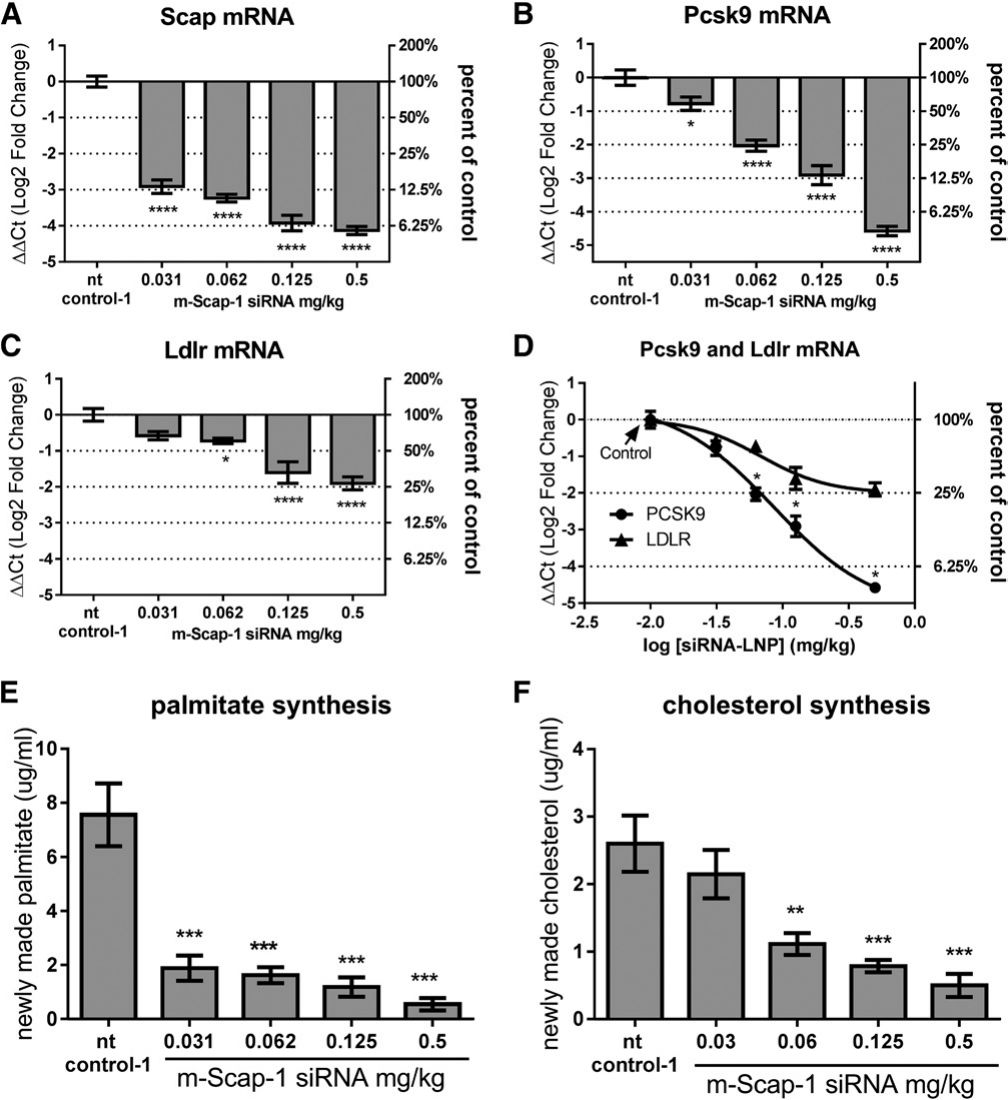
KD of liver Scap mRNA and reduced SREBP activity in mice following Scap siRNA dosing. C57BL/6 mice were dosed with the indicated siRNAs. On day 10 after siRNA dosing, mice were injected with D_2_O and livers and plasma were collected after 4 h. Scap mRNA (A), Pcsk9 mRNA (B), and Ldlr mRNA (C) levels in liver. D: Direct comparison of Pcsk9 and Ldlr mRNA levels as a function of SCAP siRNA dose. De novo palmitate (E) and cholesterol synthesis (F) were measured as described in the Materials and Methods. Each bar represents mean ± SEM; n = 8 per group. *P < 0.05, **P < 0.01, ***P < 0.001, and ****P < 0.0001.

Mouse data was analyzed by one-way ANOVA with Dunnett’s post test, except Fig. 1D where multiple *t*-test was used with statistical significance determined using the Holm-Sidak method with α = 0.05. Bar graphs represent the mean ± SEM, whereas the box and whiskers graphs represent the minimum, the 25th percentile, the median, the 75th percentile, and the maximum. All mouse data were analyzed using GraphPad Prism version 7.00 for Windows (GraphPad Software, San Diego CA).

For the longitudinal NHP studies, the data come in the format of repeated measures, for which it has become standard to quantify the mean profiles of treatments and both variation between-subject and variation within-subject over time, using a linear mixed-effects model (26): Y = Time × Treatment + ID + error. Here Time × Treatment is the mean response over time for each of the four groups separately (time and treatment interaction). It is the fixed effect for this linear mixed effects model. ID is the random effect, which characterizes inherent mean individual differences among subjects (between-subject variation). Finally, error is the residual after all fixed and random effects are removed. It is also known as the within-subject error term, or within-subject variation.

To test the drug effects, we compare the mean change-from-baseline of a treated group versus that of the vehicle group using a linear contrast (combination) of Time × Treatment levels, where the mean effect and the its standard error can be estimated using an R-package “gmodels”, which was developed by Warnes et al. (https://cran.r-project.org/web/packages/gmodels/index.html). To compare the treatment effects across different biomarkers, we further characterize the treatment effect using effect size (ES), where the mean effect is divided by the SD of the population. Unlike *P* values or T statistics, ESs are independent of the sample sizes. ESs are also normalized by their standard deviations, therefore, we can compare and rank the sensitivities among biomarkers based on their ESs.

## RESULTS

### siRNA lead selection

For mouse studies, 42 siRNA oligos were screened in Hepa 1-6 cells. siRNAs were first evaluated in primary screen for maximum target mRNA KD followed by assessment of siRNA potency in 10-point dose response experiments. As indicated in **Table 3**, four sequences were identified with greater than 3 ddCt (>85%) mRNA KD and sub-nanomolar IC50 values. All four oligos were scaled-up and formulated for evaluation in C57BL/6 mice. Assessment of hepatic Scap KD was done on days 1 and 3 post siRNA-LNP dosing. m-Scap-1 and mScap-3 siRNAs were comparable in terms of in vitro and in vivo KD levels, as shown in Table 3. m-Scap-1 was selected for further in vivo qualification and was evaluated for duration of in vivo silencing (see section below).

**TABLE 3.** Summary of in vitro and in vivo activity of mouse Scap siRNAs

siRNA Oligo	Activity in Primary Screen [KD (ddCt)]	Activity in Dose Response Experiment		Initial In Vivo Study		Repeat In Vivo Study [Day 1 KD (ddCt)]
		KD (ddCt)	IC50 (nM)	Day 1 KD (ddCt)	Day 3 KD (ddCt)	
m-Scap-1	4.1	3.8	0.15	3.9	3.3	4.0
m-Scap-2	4.3	3.8	0.15	2.2	1.9	—
m-Scap-3	5.8	4.5	0.05	3.8	3.5	3.7
m-Scap-4	3.9	3.2	0.06	3.7	3.3	—

For NHP experiments, 84 siRNA oligos were screened in rhesus MK2D cells. siRNA activity was evaluated in primary screen for maximum silencing activity followed by two rounds of dose-response experiments and assessment of duration of silencing activity. A summary of in vitro characterization of two rhesus SCAP leads is shown in **Table 4**.

**TABLE 4.** Summary of in vitro activity of NHP SCAP siRNAs

siRNA Oligo	Activity in Primary Screen [KD (ddCt)]	Activity in Dose Response Experiment 1		Activity in Dose Response Experiment 2		Duration of Silencing	
		KD (ddCt)	IC50 (pM)	KD (ddCt)	IC50 (pM)	Day 1 KD (ddCt)	Day 5 KD (ddCt)
NHP SCAP-1	5.5	6.3	9.1	7.0	7.0	6.0	6.6
NHP SCAP-2	5.0	6.5	7.0	6.9	5.4	6.0	6.8

### Liver SCAP KD in mouse dose-dependently affects Scap mRNA and SREBP pathway activity

In vivo qualification of mouse Scap siRNA was performed in C57BL/6 mice (n = 4 per group). In order to assess the magnitude and duration of KD of Scap mRNA in the liver, animals were dosed with a single dose of m-Scap-1 siRNA-LNP and liver samples collected at different time points. As shown in supplemental Fig. S1A, greater than 90% mRNA KD was observed following a single dose of m-Scap-1 siRNA. This degree of silencing was maintained over a period of 3 weeks and mRNA levels were reduced by 75% at day 28 post dosing. In agreement with the earlier reports for this LNP formulation (15), siRNA-LNP treatment was well tolerated based on the assessment of overall animal behavior and different clinical chemistry parameters, including liver and kidney function indicators (data not shown).

A siRNA-LNP dose-titration study was performed in C57BL/6 mice (n = 4 per group) to select doses covering a range of liver Scap mRNA KD. As shown in supplemental Fig. S1B, m-Scap-1 siRNA-LNP treatment with doses ranging from 0.0625 to 1 mg/kg caused a significant and dose-dependent reduction in liver Scap mRNA. The KD of liver Scap mRNA also resulted in a dose-dependent reduction of the SREBP-2 target gene, HMG-CoA reductase (Hmgcr), as well as the SREBP-1c target gene, Fasn, indicating SCAP loss of function (supplemental Fig. S1C, D). The effects on Scap, Hmgcr, and Fasn mRNA reached a maximum at the 0.5 mg/kg dose, and doses of 0.03125, 0.0625, 0.125, and 0.5 mg/kg were selected for further studies.

The dose-dependent effect of m-Scap-1 siRNA-LNP on Scap mRNA and SREBP activity was further explored in a follow-up study (n = 8 per group). The siRNA-LNP reagents did not cause any obvious toxic effects on liver, as measured by AST/ALT levels, at any of the doses tested and there was no effect on body weight in any of the treatment groups (data not shown). Scap mRNA was reduced by a maximum of 95% for the 0.5 mg/kg dose on day 10 post dosing (**Fig. 1A**). Pcsk9 and Ldlr mRNA levels were both reduced by Scap mRNA KD as shown in Fig. 1B, C. Of note, there was a differential effect of Scap mRNA KD on Pcsk9 and Ldlr levels, with Pcsk9 mRNA generally showing a more dramatic reduction relative to Ldlr mRNA (Fig. 1D).

To further demonstrate that siRNA-mediated KD of Scap resulted in loss of SREBP pathway activity, mice were dosed with D_2_O to measure palmitate and cholesterol synthesis. Consistent with the SCAP’s role in modulating SREBP-1c and SREBP-2, Scap mRNA KD caused a reduction in both palmitate and cholesterol synthesis. The effects were dose dependent with a greater percent reduction of de novo fatty acid synthesis relative to cholesterol synthesis (Fig. 1E, F).

### Effects of Scap mRNA KD on plasma lipids, Pcsk9, and liver LDLR protein in mice

**Fig. 2.**
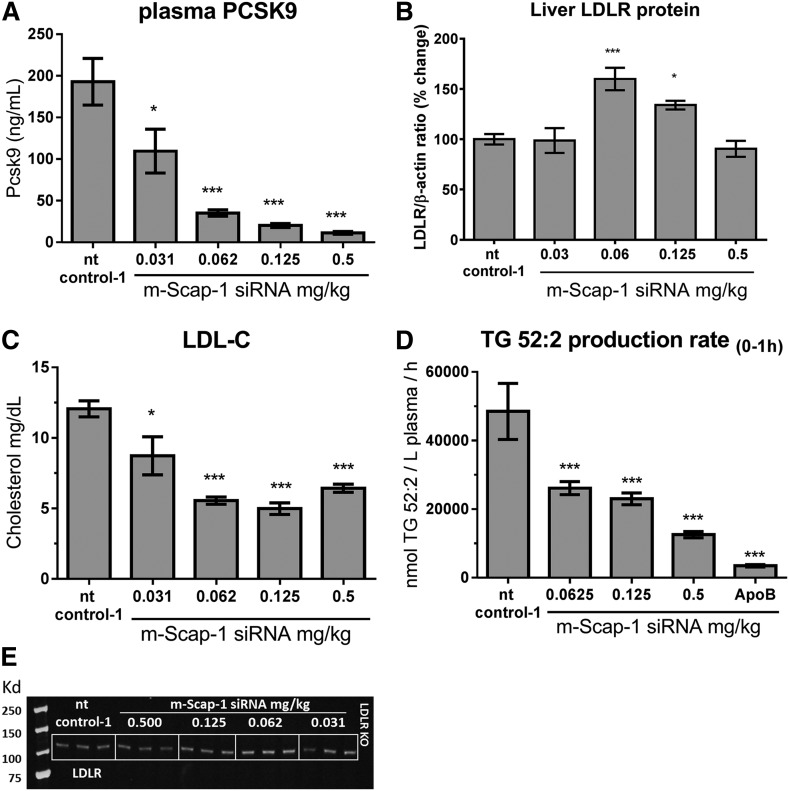
Effect of Scap siRNA on plasma PCSK9, LDL-C, TG production, and LDLR protein. C57BL/6 mice were dosed with the indicated siRNAs and plasma and livers were collected 10 days after dosing. PCSK9 (A) and LDL-C (C) were measured in EDTA-plasma. B: Quantification of LDLR protein as measured by Western blot. D: TG production rate was measured in plasma by LC-MS/MS after an intravenous dose of [^13^C_18_]oleic acid as described in the Materials and Methods. E: Representative Western blot of liver LDLR protein. Each bar represents mean ± SEM; n = 8 per group [except (B), n = 6 per group]. *P < 0.05 and ***P < 0.001.

Liver Scap mRNA KD caused a significant reduction in total plasma cholesterol at all siRNA-LNP doses. Plasma TG was significantly reduced at the 0.125 and 0.5 mg/kg siRNA-LNP doses (supplemental Fig. S2). Dose-dependent reductions were also observed for plasma total cholesterol, LDL-C, VLDL-C, and HDL-C (**Fig. 2C**; supplemental Fig. S2B–D; supplemental Fig. S5). To explore the mechanism of lipid lowering, we measured the levels of plasma PCSK9, liver LDLR protein level, and TG synthesis + secretion rate. As shown in Fig. 2A, plasma PCSK9 was dramatically and dose-dependently reduced by siRNA-mediated Scap KD. Plasma PCSK9 was reduced by a maximum of 94% at the 0.5 mg/kg siRNA-LNP dose on day 10 after injection (Fig. 2A). Interestingly, the liver LDLR protein-Scap siRNA dose-relation showed a bell-shaped curve with a significant increase in LDLR protein levels at the 0.0625 and 0.125 mg/kg doses, whereas the 0.5 mg/kg dose showed no significant change (Fig. 2B, E). This bell-shaped dose response curve likely contributed to an observed U-shaped Scap siRNA-LNP dose-response effect on LDL-C (Fig. 2C). To determine whether a reduction in VLDL secretion contributed to the reduced plasma lipid levels, the TG production rate following intravenous administration of [^13^C_18_]oleic acid was measured. We have previously demonstrated using this protocol in mice that the majority of newly made TG appearing in plasma is contained in VLDL and thus measurements made in total plasma should be a reasonable surrogate to determine VLDL-TG production (27). Treatment with m-Scap-1 siRNA resulted in a dose-responsive reduction in the appearance of newly made TG in plasma and was most dramatic at 0.5 mg/kg dose (Fig. 2D; supplemental Fig. S3).

### SCAP inhibition and the statin treatment have additive effects on LDL-C reduction in NFR-CETP mice

**Fig. 3.**
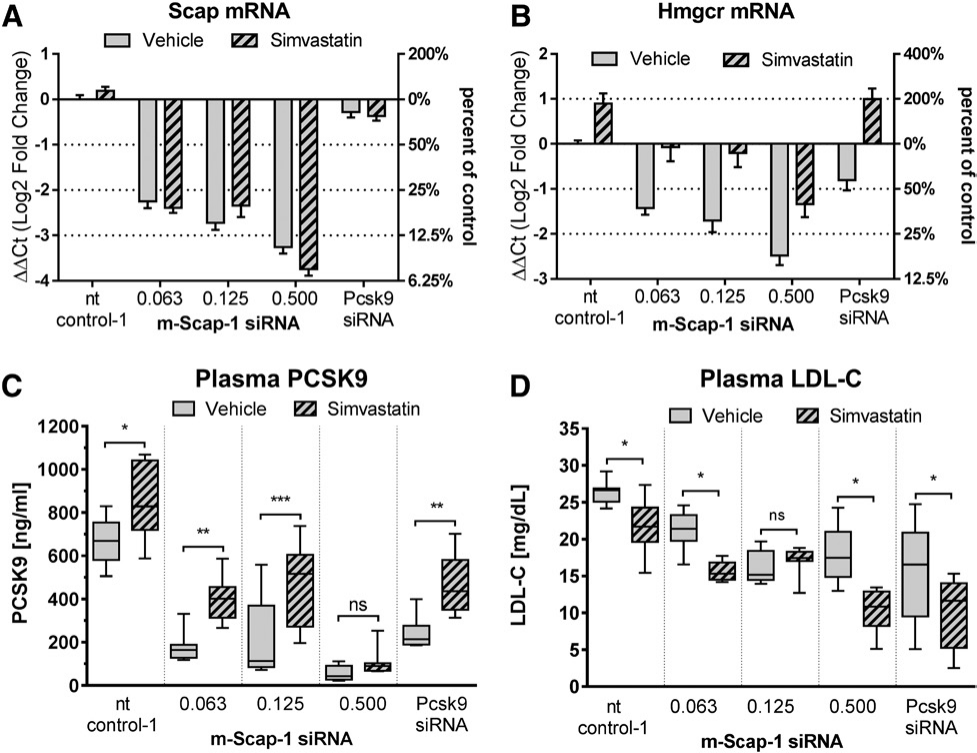
Scap siRNA KD adds to simvastatin LDL-C lowering in NFR-CETP mice. Mice were dosed orally daily with 100 mg/kg simvastatin or vehicle starting on day 0. On day 4, mice were dosed with nt control-1 siRNA, Pcsk9 siRNA (0.5 mg/kg), and m-Scap-1 siRNA at the indicated doses (milligrams per kilogram). Plasma and livers were collected 10 days later. Liver Scap mRNA (A), liver Hmgcr mRNA (B), plasma PCSK9 (C), and plasma LDL-C (D). Each bar in (A) and (B) represents mean ± SEM. Box and whiskers in (C) and (D) represent the 25th percentile, the median, the 75th percentile (boxes), and the minimum and maximum (whiskers); n = 8 per group. *P < 0.05, **P < 0.01, and ***P < 0.001.

The statin class of drugs inhibits HMGCR, the rate-limiting enzyme in cholesterol biosynthesis, thereby reducing cholesterol synthesis and free cholesterol in the cell. In the liver, the reduction in free cholesterol leads to a compensatory upregulation of SREBP activity, including upregulation of LDLR and PCSK9 expression levels. In humans, a net increase in LDLR activity and LDL-C lowering is the result of statin treatment (28). To test to determine whether Scap KD prevents the ability of a statin to increase SREBP activity, we pretreated NFR-CETP mice with simvastatin followed by Scap KD using different doses of m-Scap-1 siRNA. NFR-CETP mice were selected for this study because this strain of mice has a higher level of LDL-C relative to wild-type mice, allowing testing of whether statin treatment and Scap KD would have an added benefit on LDL-C levels. Scap KD was equally effective in simvastatin- and vehicle-treated mice (**Fig. 3A**). As shown in Fig. 3B, simvastatin treatment upregulated Hmgcr expression by approximately 2-fold relative to vehicle treatment in mice also dosed with control and Pcsk9 siRNA, indicating SREBP transcriptional activation. As seen in C57BL/6 mice, Scap siRNA dose-dependently reduced Hmgcr mRNA levels in NFR-CETP mice. Simvastatin increased Hmgcr levels in mice treated with Scap siRNA by at least 2-fold, although the levels were still lower relative to the control siRNA-treated mice (Fig. 3B). Simvastatin also increased circulation of Pcsk9 in all treatment groups (Fig. 3C), demonstrating that sufficient SCAP activity remained to allow for SREBP activation. As shown in Fig. 3D, siRNA-mediated KD of Scap and Pcsk9 in NFR-CETP mice reduced LDL-C levels in the non-statin-treated (vehicle) groups (gray boxes). Of interest, simvastatin treatment (hatched boxes) further reduced LDL-C levels in all siRNA groups (except in the 0.125 mg/kg Scap siRNA group), suggesting that both Pcsk9 KD and Scap KD and the statin mechanism have additive effects on LDL-C reduction in this mouse model.

### siRNA-mediated KD of SCAP in rhesus monkey reduces SREBP gene expression, de novo lipogenesis, and circulating PCSK9 and lipid levels

siRNA lead qualification was done in lean rhesus monkeys with two independent SCAP siRNA leads. Animals (n = 4) were dosed with a single dose of SCAP, PCSK9, or nt control (nt control-2) siRNAs and mRNA levels assessed from liver biopsies obtained 4 days prior to dosing and on days 7, 14, and 28 following siRNA administration (supplemental Fig. S4A). Both SCAP siRNAs showed over 80% mRNA KD in the liver at day 7. SCAP KD was associated with significant reduction in plasma PCSK9 levels (supplemental Fig. S4B). Maximum plasma PCSK9 reduction was close to 75% for the first 9 days following siRNA dosing. Based on these results, NHP SCAP-1 siRNA was selected as the lead for the follow up studies.

**Fig. 4.**
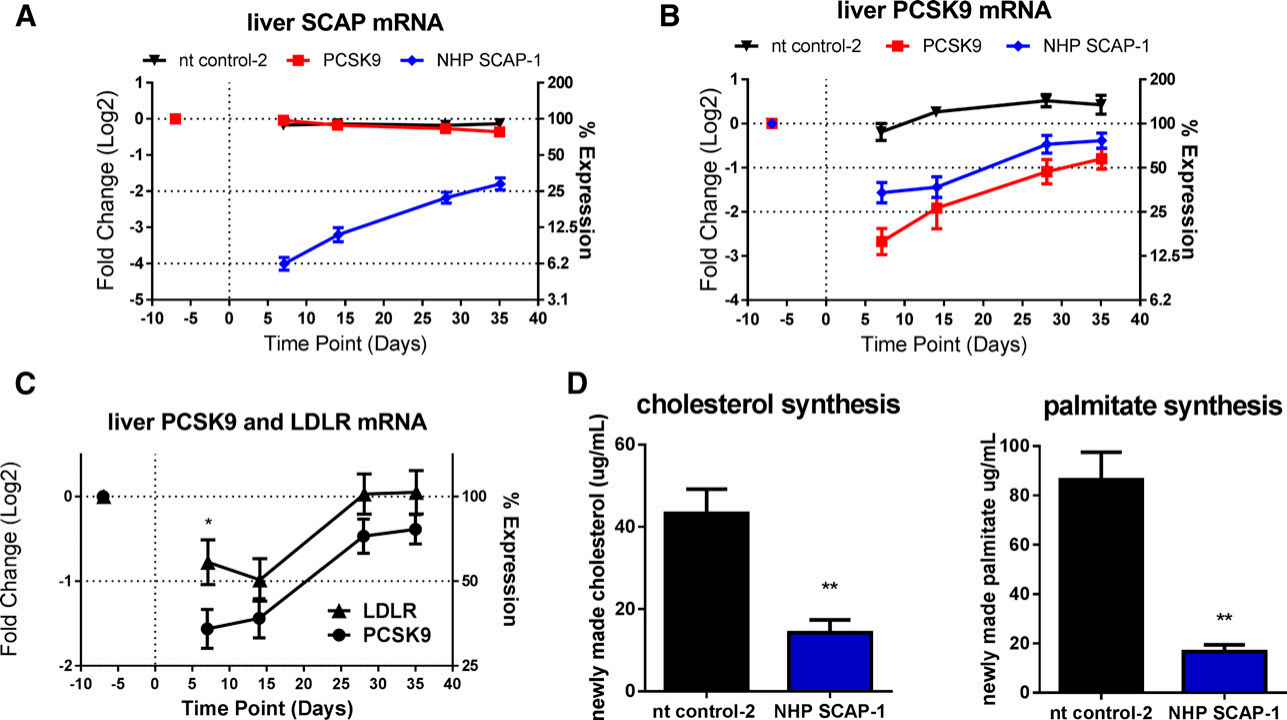
Effect of single dose SCAP siRNA on SCAP mRNA and SREBP activity in rhesus macaque monkeys. Lean rhesus monkeys were dosed intravenously with the indicated siRNAs. Liver biopsy samples were obtained on day −7 (prior) and days 7, 14, 28, and 35 after siRNA dosing for measurement of liver SCAP mRNA (A), liver PCSK9 mRNA (B), and liver LDLR mRNA. Figure 4C shows a direct comparison of liver PCSK9 and LDLR mRNA. D: On day 16, animals were administered D_2_O and blood samples were collected at −0.5, +4, +24, and +48 h for the determination of palmitate and cholesterol synthesis. Each symbol or bar represents mean ± SEM; n = 4–10 per group. Liver SCAP mRNA was significantly lower in the NHP SCAP-1 group relative to nt control-2 at all time points (*P* < 0.05). Liver PCSK9 mRNA was lower in the PCSK9 and NHP SCAP-1 groups relative to nt control-2 group at all time points (*P* < 0.05). *P < 0.05 and **P < 0.01.

**Fig. 5.**
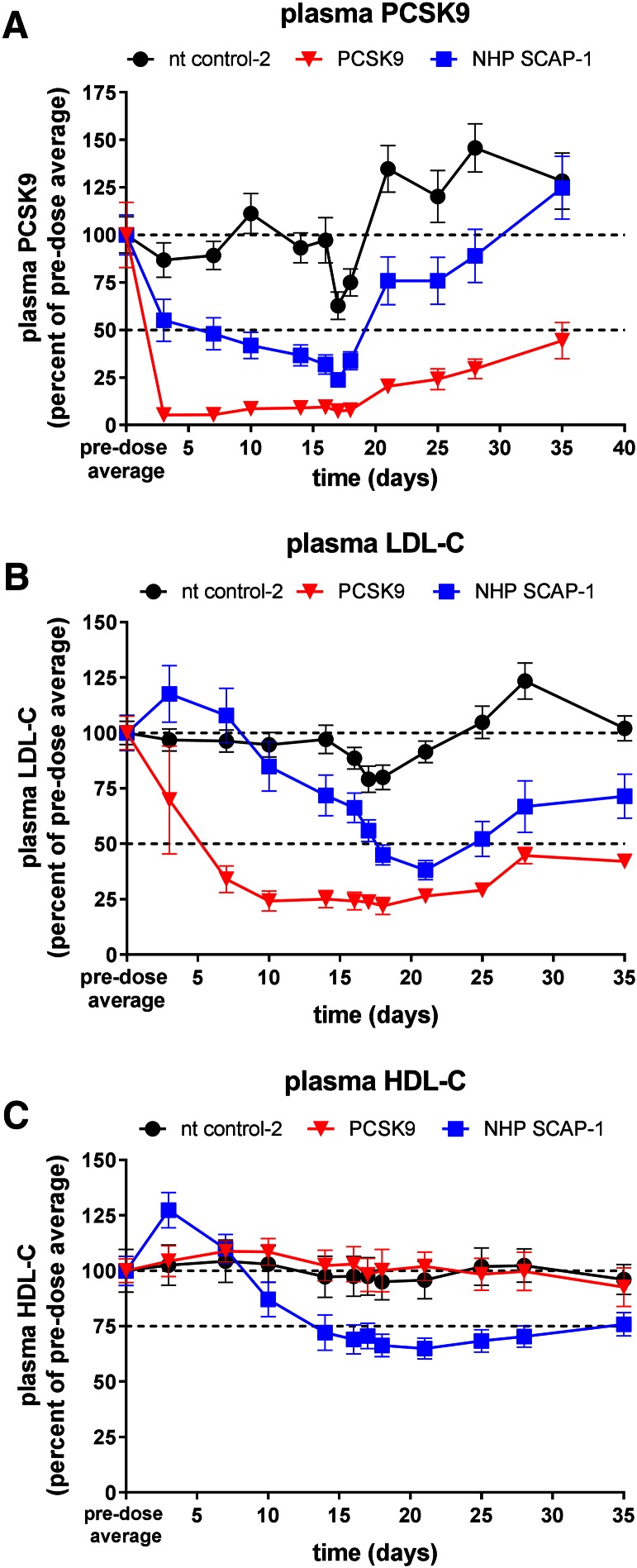
Reduction in plasma PCSK9 and lipids in rhesus monkeys after treatment with SCAP siRNA. Lean rhesus macaque monkeys were dosed intravenously with the indicated siRNAs on day 0 and blood was collected for EDTA plasma on several days for analysis as indicated in the graphs. Plasma PCSK9 (A), plasma LDL-C (B), and plasma HDL-C (C). PCSK9 was significantly lower in the NHP SCAP-1 siRNA group relative to nt control-2 on days 3–21 and significantly lower in the PCSK9 siRNA group relative to nt control-2 on days 3–35 (*P* < 0.05). LDL-C was significantly lower in the NHP SCAP-1 siRNA group relative to nt control-2 on days 14 and 18–35, and significantly lower in the PCSK9 siRNA group relative to nt control-2 on days 3–35 (*P* < 0.05). HDL-C was significantly lower in the NHP SCAP-1 siRNA group relative to nt control-2 on days 10–35 (*P* < 0.05). Each symbol represents mean ± SEM; n = 4–10 per group.

To further characterize the effect of hepatic SCAP KD on SREBP activity, including de novo lipogenesis, a follow up study was performed with a larger group size and addition of the D_2_O tracer on day 16 post siRNA-LNP dosing. Animals were dosed with a single dose of SCAP, PCSK9, and nt control (nt control-2) siRNA and the expression of SCAP, PCSK9, and LDLR mRNA was measured in liver biopsies 7 days before and 7, 14, 28, and 35 days post siRNA-LNP dosing. SCAP mRNA was reduced by 94% on day 7 relative to a predose liver biopsy. The expression of SCAP gradually increased over time as shown in Fig. 4A. PCSK9 and the nt control did not change the SCAP expression levels (Fig. 4A). SCAP and PCSK9 siRNA both reduced liver PCSK9 mRNA levels (Fig. 4B). The effect of SCAP KD on PCSK9 and LDLR mRNA is shown in Fig. 4C. The effect of SCAP siRNA on PCSK9 mRNA was more pronounced relative to the effect on LDLR mRNA similar to what was observed in the mouse studies. Both cholesterol and palmitate de novo lipogenesis were significantly reduced by 67 and 81%, respectively, relative to control siRNA (Fig. 4D). Consistent with the reduced PCSK9 mRNA levels, SCAP siRNA treatment caused a significant and robust reduction in plasma PCSK9 levels (**Fig. 5A**). The PCSK9 siRNA-treated groups also showed the expected reduction in plasma PCSK9 levels. Analysis of plasma lipid levels revealed a very significant reduction of LDL-C in the SCAP siRNA-treated group that was similar in magnitude to the reduction in the PCSK9 siRNA group on day 21 of the study (Fig. 5B). HDL-C was also reduced in the SCAP siRNA-LNP treatment group, whereas the nt control and PCSK9 siRNA did not affect HDL-C levels (Fig. 5C). These data demonstrate that SCAP loss of function in a higher species leads to a dramatic suppression of SREBP pathway activity similar to what has been shown in rodent models.

### Dose-dependent effects of SCAP siRNA on SREBP pathway activity and plasma lipids in NHP

**Fig. 6.**
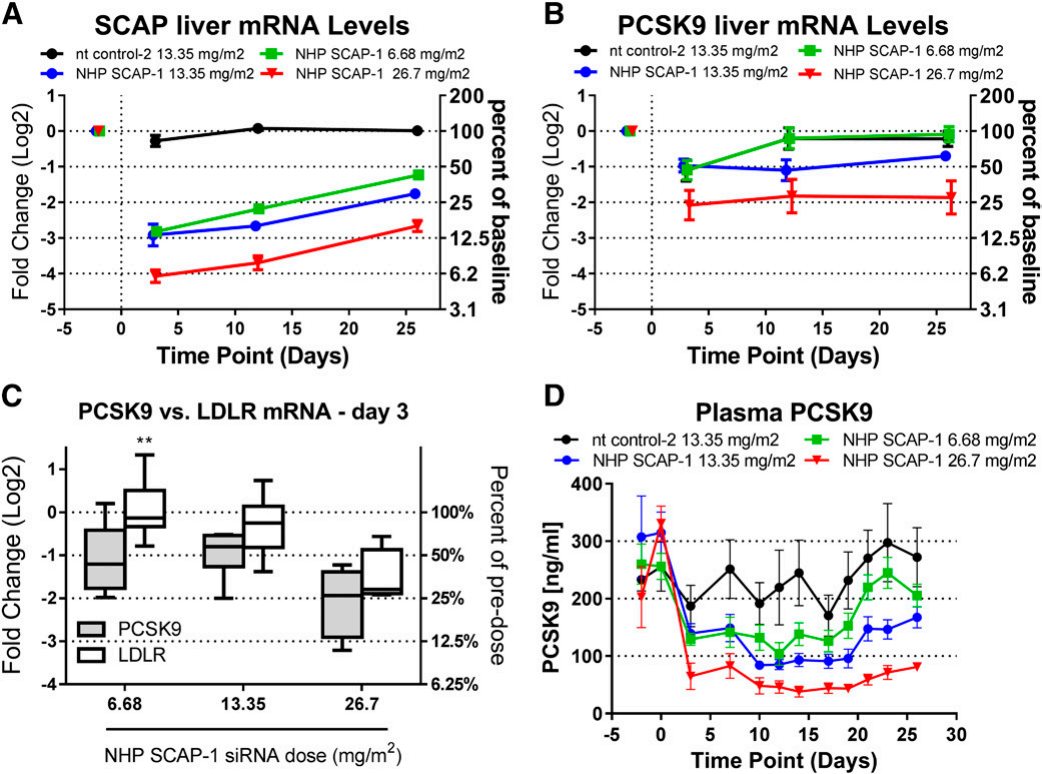
Dose-dependent reduction in liver SCAP, PCSK9 mRNA, and plasma PCSK9 in rhesus monkeys following treatment with SCAP siRNA. Lean rhesus macaque monkeys were dosed intravenously with the indicated siRNAs on day 0 and blood was collected for analysis on the days indicated in the graphs. Liver biopsy samples were obtained on day −2 (prior) and days 3, 12, and 26 after siRNA dosing for measurement of liver SCAP mRNA (A) and liver PCSK9 mRNA (B). C: Comparison of liver PCSK9 and LDLR mRNA on day 3 for the indicated NHP SCAP-1 siRNA doses. D: Plasma PCSK9 levels. Each symbol represents mean ± SEM. Box and whiskers in (C) represent the 25th percentile, the median, the 75th percentile (boxes), and the minimum and maximum (whiskers). Liver SCAP mRNA was significantly reduced (*P* < 0.05) for all doses and time points relative to nt control-2. Liver PCSK9 mRNA was significantly lower in the 26.7 mg/m^2^ dose group relative to nt control-2 at all time points and significantly lower in the 13.35 mg/m^2^ dose group relative to nt control-2 on day 12 (*P* < 0.05). Plasma PCSK9 was significantly reduced for all SCAP siRNA doses relative to nt control-2 siRNA on days 3–28 (*P* < 0.05) (n = 4 for the 26.7 mg/m^2^ dose group; n = 8 for all other groups).

**Fig. 7.**
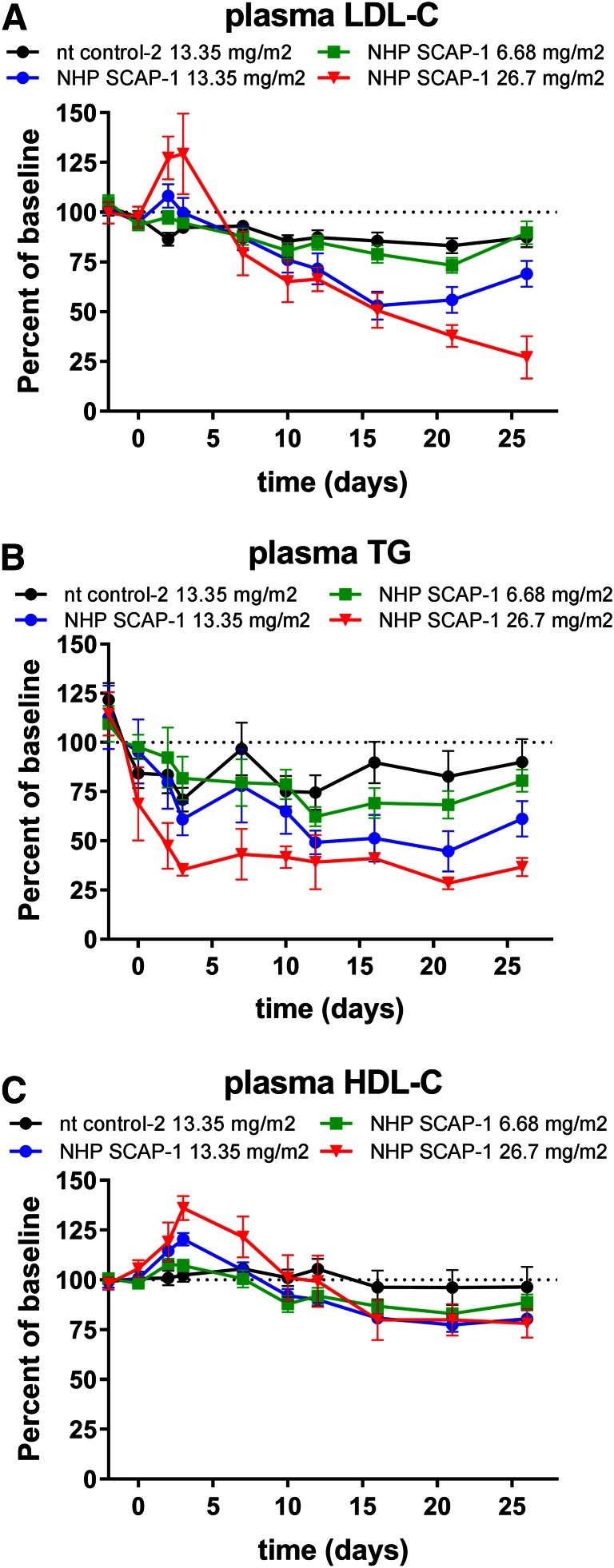
Dose-dependent reduction in plasma lipids in rhesus monkeys following treatment with SCAP siRNA. Lean rhesus macaque monkeys were dosed intravenously with the indicated siRNAs on day 0 and blood was collected for EDTA plasma on several days for analysis as indicated in the graphs. Plasma LDL-C (A), plasma TG (B), and plasma HDL-C (C). Each symbol represents mean ± SEM; n = 4–8 per group. Plasma LDL-C and TG levels were significantly lower in the 26.7 mg/m^2^ dose group relative to nt control-2 on days 10–26 and significantly lower in the 13.35 mg/m^2^ dose group on days 16–26 (*P* < 0.05). Plasma HDL-C was significantly lower in the 13.35 mg/m^2^ dose group relative to nt control-2 on days 21 and 26 (*P* < 0.05).

To determine the dose-dependent effects of SCAP mRNA KD on SREBP pathway activity, rhesus monkeys were dosed with 6.68, 13.35, and 26.7 mg/m^2^ of the SCAP siRNA-LNP reagent. The nt siRNA-LNP was dosed at 26.7 mg/m^2^ as a control. Liver SCAP mRNA was dose-dependently reduced by the escalating doses of SCAP siRNA at all time points measured (**Fig. 6A**). Liver PCSK9 mRNA and plasma PCSK9 levels were significantly reduced and showed a dose-dependent correlation with the SCAP siRNA dose (Fig. 6B, D). On day 3 after siRNA dosing, the effect of SCAP mRNA KD on PCSK9 mRNA was more pronounced relative to LDLR mRNA for all siRNA doses tested and reached statistical significance for the low dose (Fig. 6C). As shown in **Fig. 7**, plasma LDL-C was significantly reduced for the 13.35 mg/m^2^ dose and plasma TG levels were significantly reduced at the 13.35 and 26.7 mg/m^2^ dose level. LDL-C showed a trend toward reduction at the 26.7 mg/m^2^ level, but this did not meet statistical significance. There was a reduction in HDL-C levels, although less pronounced relative to what was observed in the single-dose siRNA study.

### Effects of siRNA-mediated KD of SCAP on liver gene expression in rhesus monkey

**Fig. 8.**
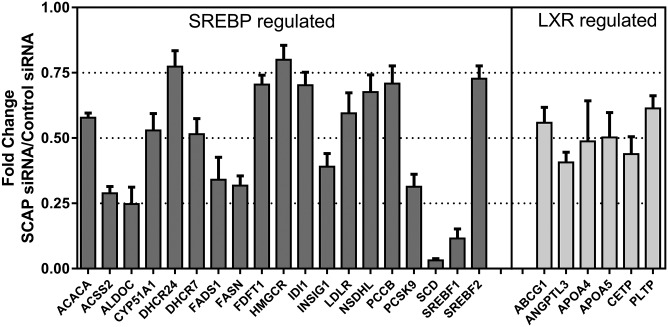
Fold changes in SREBP- and LXR-regulated gene expression after SCAP liver KD in rhesus monkeys. A panel of 372 genes involved in lipid and glucose metabolism were assayed in the livers of SCAP- and control siRNA-treated rhesus monkeys using a custom gene expression array. Representative data are shown as fold change expression in the SCAP siRNA treatment group relative to the control siRNA-treated group. All data *P* < 0.05. The full data set is shown in supplemental Table S1. Each bar represents mean ± SEM; n = 9–10 per group.

The expression of a selection of genes involved in liver lipid and glucose metabolism pathways were measured in the liver samples collected from the rhesus monkeys on day 14 after a single dose of SCAP siRNA. The gene expression in the samples from SCAP siRNA-treated animals was normalized to the nt control-treated animals at the same time point. A custom gene expression array was used in the analysis (24). Consistent with data from rodent studies, SREBP-regulated genes were downregulated in response to SCAP siRNA treatment in the liver of rhesus monkeys (**Fig. 8**, supplemental Table S1). The most dramatic changes were found for SREBP-regulated genes encoding enzymes involved in fatty acid synthesis, consistent with the reduction of fatty acid synthesis (Fig. 4). Genes encoding SREBP-2 and enzymes involved in cholesterol synthesis were also significantly downregulated. In addition, the gene expression analysis revealed the downregulation of several LXR and PPARα target genes (Fig. 8, supplemental Table S1).

## DISCUSSION

SCAP is a key regulator of lipid metabolism and a potential therapeutic target for treatment of dyslipidemia and fatty liver disease (1, 2). Of particular interest for this study was to determine the effect of SCAP KD on LDL-C in circulation. Plasma LDL-C levels are regulated by a number of factors, including VLDL secretion rate, conversion of VLDL to LDL particles, and the clearance of LDL particles by the LDLR (29). SCAP affects these factors by regulating de novo lipogenesis and thereby VLDL secretion and by regulating the expression of LDLR and PCSK9 at the mRNA level. The effect of SCAP KD on VLDL secretion and PCSK9 should lead to a reduction in LDL-C; however, these effects could potentially be counteracted by the direct effect of SCAP KD on LDLR expression.

In mice, Scap KD resulted in dose-dependent reduction in the expression of SREBP-regulated genes, de novo lipogenesis, TG production rate, plasma PCSK9, and plasma lipids. PCSK9 expression was highly regulated by Scap via inhibition of SREBP-2, as Scap KD led to a dramatic reduction in both the mRNA and plasma protein levels. Ldlr mRNA was less sensitive to Scap KD relative to Pcsk9 mRNA at all Scap siRNA doses tested (Fig. 1). PCSK9 interacts with LDLR on the surface of cells to cause its internalization and degradation (30). The counteracting effects of Scap KD on Ldlr mRNA reduction and the reduction in plasma PCSK9 led to a nonlinear dose effect of Scap KD on LDLR protein in the liver. Two doses of Scap siRNA increased LDLR protein levels relative to the siRNA control group; whereas, at the lowest and highest doses, there was no significant change in LDLR. This was mirrored by the U-shaped dose-response effect on LDL-C, suggesting that the changes in LDLR protein levels reflected LDLR activity as measured by plasma LDL-C levels. Thus, in mice, Scap KD affected the major factors regulating LDL-C: VLDL secretion (measured by TG production rate), plasma PCSK9 protein levels, and LDLR levels. All doses of Scap siRNA reduced LDL-C, although the dose-response was nonlinear and the maximum effect was achieved at a dose that did not maximize Scap KD and function. Plasma PCSK9, VLDL-C, and TG all showed a more linear correlation with Scap mRNA KD.

Statins are an important class of cholesterol-lowering drugs extensively used in the prevention of cardiovascular disease. Statins inhibit HMGCR, a rate limiting enzyme in cholesterol synthesis, and reduce cellular cholesterol levels. In response, SREBP is released from the ER membrane and cleaved in the Golgi by proteases allowing translocation to the nucleus where SREBP-responsive genes are induced, including the LDLR and HMGCR. LDL-C levels are reduced as a consequence of higher LDLR expression and hepatic cholesterol levels reestablished (28). To test how Scap KD would interact with the statin effect on SREBP activity in mice, we combined Scap siRNA dosing and simvastatin treatment in NFR-CETP mice. We have previously identified the NFR-CETP model as a sensitive responder model to statin treatment relative to C57BL/6 and a diet-induced obesity model based on SREBP pathway gene expression changes (data not shown). Simvastatin induced both Hmgcr mRNA expression and circulating PCSK9 relative to the vehicle-treated mice in all siRNA-treated mice (control, Scap, and Pcsk9 siRNA treatment groups). Although the levels of Hmgcr mRNA and circulating PCSK9 were lower in the statin + Scap siRNA-treated groups relative to vehicle + control siRNA-treated mice, these data demonstrate that inhibition of the HMGCR enzyme and SREBP activation was achieved in the NFR-CETP mice even in the context of Scap mRNA KD. Simvastatin and Pcsk9 siRNA treatment individually reduced LDL-C in NFR-CETP mice (Fig. 3D), and in combination, the effect on LDL-C was more pronounced. The additive effect was expected as Pcsk9 mRNA KD amplifies the effect of statin on LDLR induction in mice (31). Scap KD and statin treatment also showed an additive effect on LDL-C lowering for two of the three doses tested. This additive effect could be due to the PCSK9 reduction observed in the Scap siRNA-treated mice or suppression of VLDL secretion. More detailed studies are needed to determine the exact mechanism.

The effects of SCAP loss of function have been studied extensively in mouse and hamster. The lipid metabolism pathways of these models may not fully translate into human (14). Therefore, to examine the complex effects of SCAP KD on plasma lipids, we performed SCAP siRNA KD studies in rhesus monkeys. PCSK9 siRNA was used as a positive control. SCAP siRNA reduced SCAP mRNA in the liver by 94% on day 7 relative to a predose followed by a gradual recovery toward baseline as the siRNA was cleared from the liver. The KD of SCAP had a profound effect on SREBP activity: cholesterol and palmitate synthesis were significantly reduced, as were liver mRNA and circulating PCSK9 levels. These effects were SCAP siRNA dose-dependent. Similar to the observation in mice, the degree of PCSK9 mRNA suppression was larger than that of the LDLR mRNA both in the single dose (Fig. 4) and in the dose titration studies (Fig. 6). In rhesus monkeys, SCAP KD led to a dramatic reduction in circulating PCSK9 levels that approached levels of reduction observed in the PCSK9 siRNA-treated group. Importantly, plasma LDL-C levels were robustly reduced by SCAP KD in both studies by more than 50%. The effect on LDL-C was slower to manifest in the SCAP siRNA group compared with the PCSK9 siRNA group. This difference could be partially due to a lag in SCAP protein turnover, delaying the effects on SREBP transcriptional activity or could reflect the potentially more complex mechanism of LDL-C reduction mediated by SCAP loss of function relative to PCSK9 KD. Plasma TG levels were significantly reduced suggesting that the reduced de novo synthesis of cholesterol and palmitate caused a reduction in lipoprotein particle secretion, although this was not measured directly in the current study.

Liver gene expression analysis, using a custom gene expression array, demonstrated several SREBP-regulated genes to be downregulated. Many of the genes on the array reported to be regulated predominantly by SREBP-2 in mice (including the SREBF2 gene) (2) were significantly downregulated by SCAP KD on day 14 after siRNA dosing in rhesus monkeys. Many of these genes encode enzymes involved in cholesterol synthesis and would explain the reduced cholesterol synthesis. Similarly, genes regulated predominantly by SREBP-1c in mice (including the SREBF1 gene) (2) and involved in de novo fatty acid synthesis were also downregulated, including ACC1, FASN, FADS1, and SCD1. Overall, the reduction in SREBP-1c-regulated genes was more dramatic relative to SREBP-2-regulated genes. Interestingly, LXR-regulated genes were significantly reduced as well. This could be explained by the reduced cholesterol synthesis in the liver, which in turn could reduce the levels of oxysterols, the endogenous LXR ligands. Because SREBP-1c, SCD, and other fatty acid synthesis genes are direct targets of LXR, a reduced LXR transcriptional activity would also explain the more dramatic changes in SREBP-1c-regulated genes relative to SREBP-2- regulated genes. Among the downregulated genes is ABCG1, a cholesterol efflux gene. Reduced expression of ABCG1 and potentially less free cholesterol available for efflux (due to reduced cholesterol synthesis) could have contributed to the lower plasma HDL-C levels observed in the SCAP siRNA-treated group. The altered expression of the HDL-modifying proteins, PLTP and CETP, could also have contributed to the effect. The reduction in HDL-C was most likely not caused by increased ApoE-mediated clearance by the LDLR, which has been proposed to occur in mice as a result of PCSK9 inhibition, as there was no observed lowering of HDL-C in the PCSK9 siRNA-treated rhesus monkeys. Based on the dramatic suppression of de novo fatty acid synthesis, it is conceivable that PPARα-regulated genes would be downregulated. A number of genes on the array reported to be regulated by PPARα in human cells were indeed downregulated, including ACADVL, ACSL3, SLC27A2, and G6PC (32). Other well-known PPARα-regulated genes were not changed (e.g., ACSL1, CPT1A, FABP1), suggesting that the effect of PPARα transcriptional activity was relatively modest or counteracted by the change in the activity of other transcriptional regulators.

The SCAP siRNA studies reported herein were carried out in lean normolipidemic mouse and rhesus monkey models that were fed standard relatively low-fat diets. Horton and colleagues investigated the effects of liver SCAP loss of function in ob/ob mice, mice fed a high-fat diet, and hamsters fed a high-sucrose diet and found effects on plasma lipids and lipid metabolism similar to what is reported here (8). Specifically, the differential effect of SCAP loss of function on Pcsk9 and Ldlr mRNA expression were observed in these models as well. It would be of interest to study the effects of siRNA-mediated SCAP KD on plasma lipids and lipid metabolism in dysmetabolic NHP models to further investigate the therapeutic potential of liver SCAP inhibition.

The requirement of SCAP for the activation of lipid biosynthesis could be a potential driver of toxicity in conjunction with systemic SCAP inhibition, e.g., using a small molecule inhibitor approach. Indeed, conditional knockout of SCAP in Swann cells causes hypomyelination (33) and conditional knockout of SCAP in the intestinal mucosa causes severe enteropathy (34). Due to the risk of adverse effects from systemic inhibition of SCAP, specific inhibition of SCAP in the liver is preferable for a therapeutic approach. We have previously shown that ionizable LNPs, similar to the one described here, primarily target liver in the biodistribution studies (35), suggesting that this platform could be used as a potentially suitable approach for safe and efficacious inhibition of SCAP.

In summary, we have demonstrated for the first time that SCAP siRNA-mediated KD in a NHP model leads to a reduction in plasma PCSK9, TG, and LDL-C suggesting a cardioprotective outcome of SCAP inhibition in humans. This opens up potential targeting of SCAP in the liver for the treatment of dyslipidemia and potentially other metabolic diseases that are driven by increased SREBP activation such as fatty liver disease.

## Supplementary Material

Supplemental Data
