# Supplementary material for: Dose-dependent effects of siRNA-mediated inhibition of SCAP on PCSK9, LDLR, and plasma lipids in mouse and rhesus monkey
Source: J Lipid Res. 2016 Nov 28;57(12):2150–62. doi: 10.1194/jlr.M071498 (PMC5321219; doi:10.1194/jlr.M071498)
Supplement: Supplemental Data [file supp_57_12_2150__index.html]

Dose-dependent effects of siRNA-mediated inhibition of SCAP on PCSK9, LDLR and plasma lipids in mouse and rhesus monkey — Dose-dependent effects of siRNA-mediated inhibition of SCAP on PCSK9, LDLR, and plasma lipids in mouse and rhesus monkey — Supplemental Data 

# Dose-dependent effects of siRNA-mediated inhibition of SCAP on PCSK9, LDLR, and plasma lipids in mouse and rhesus monkey

## Supplemental Data

- supplemental file (.pdf, 992 KB) - supplemental file
