# Supplementary material for: Dose-dependent effects of siRNA-mediated inhibition of SCAP on PCSK9, LDLR, and plasma lipids in mouse and rhesus monkey
Source: J Lipid Res. 2016 Nov 28;57(12):2150–62. doi: 10.1194/jlr.M071498 (PMC5321219; doi:10.1194/jlr.M071498)
Supplement: Supplemental Data [file 10.1194_M071498_jlr.M071498-1.pdf]

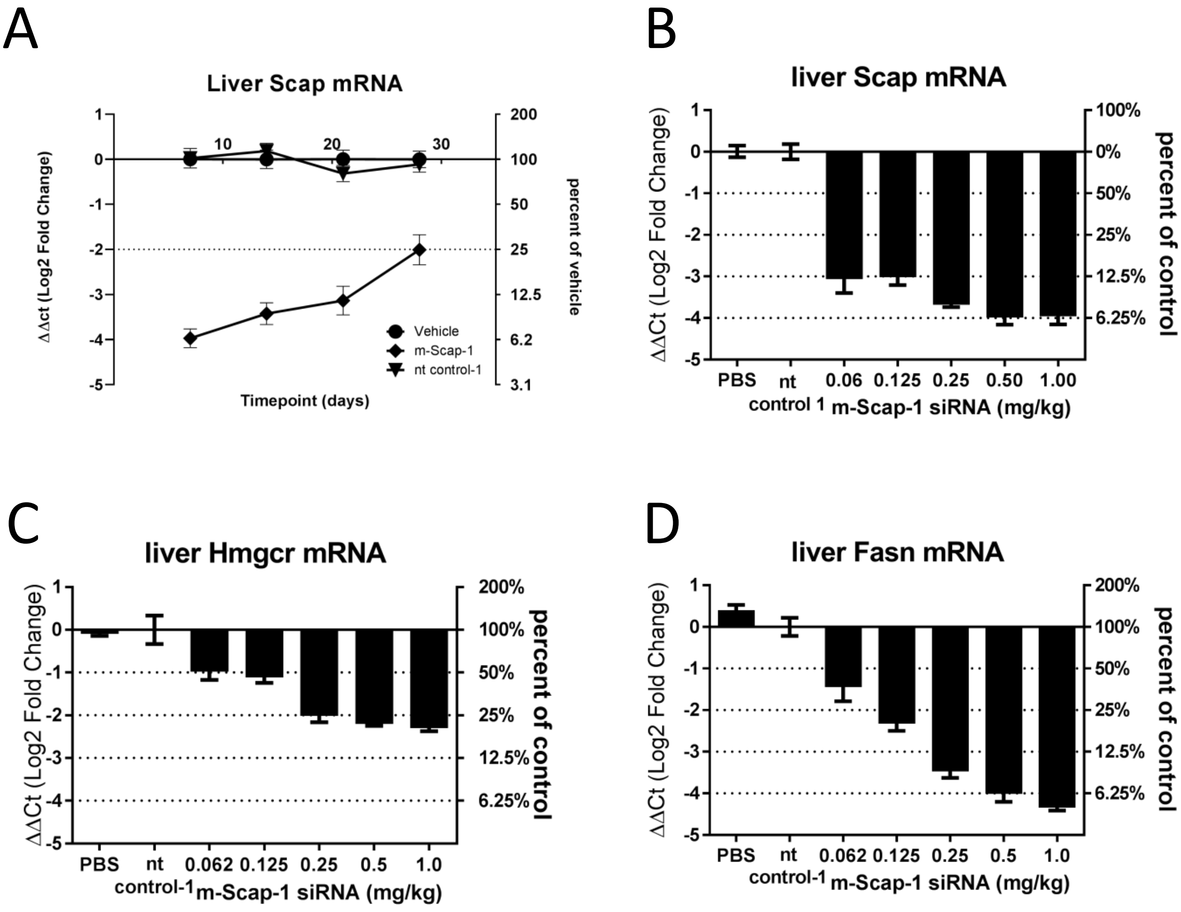

Supplemental Figure S1: Scap siRNA-LNP single dose time course and dose titration in C57BL/6 mice. (A) Mice (n=4 per time point) were injected with m-Scap siRNA and livers were collected on days 7, 14, 21 and 28 for Scap mRNA expression. (B), (C), and (D) C57BL/6 mice were dosed with the indicated doses of m-Scap siRNA. On day 10 after siRNA dosing, livers were collected and expression of SREPB regulated genes were measured by real time PCR (taqman).

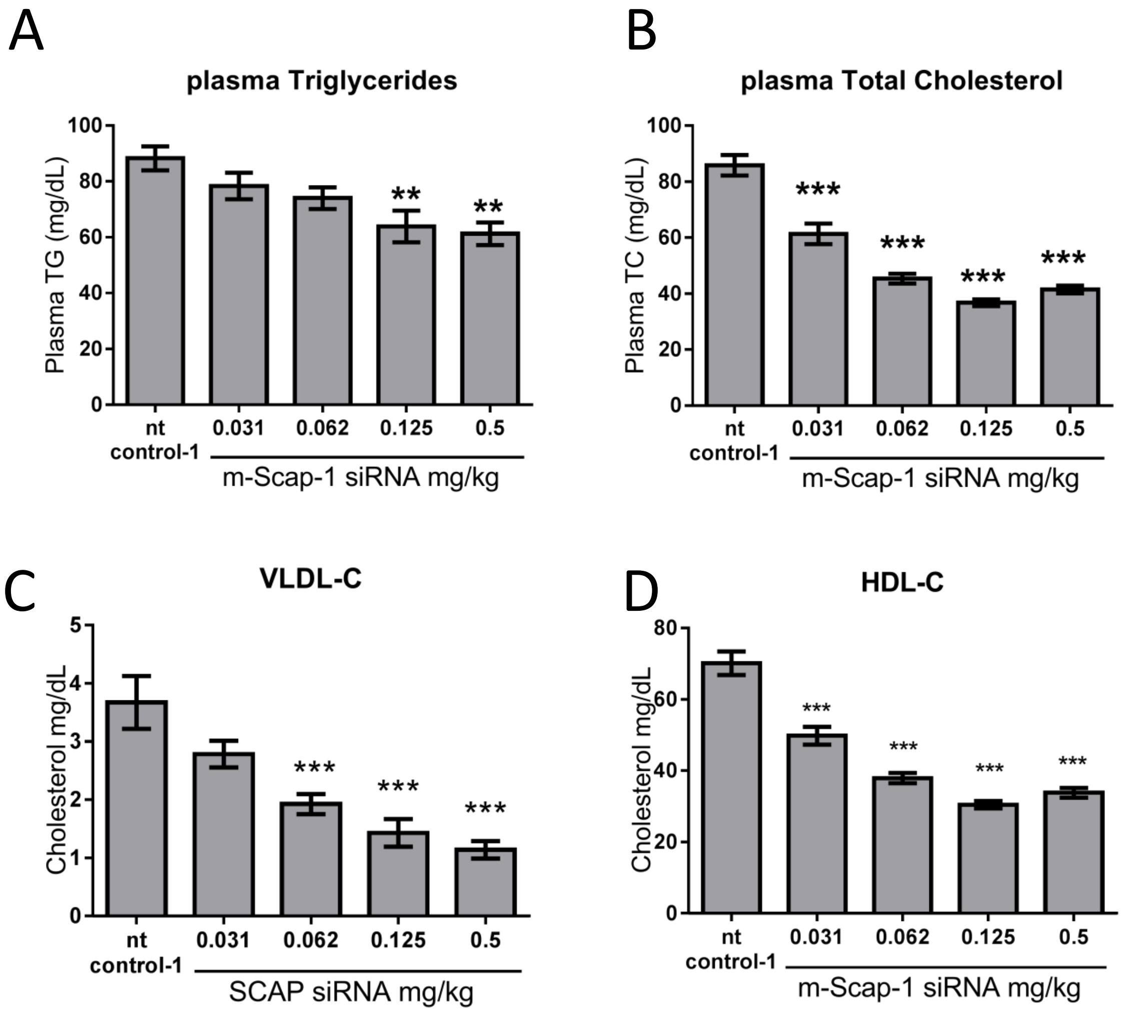

Supplemental Figure S2: Effects of Scap siRNA-LNP dose titration on plasma lipids in C57BL/6 mice. C57BL/6 mice were dosed with the indicated siRNAs and plasma was collected 10 days after dosing. (A) plasma TG, (B) plasma total cholesterol, (C) plasma VLDL-C and (D) plasma HDL-C were measured in EDTA-plasma. Each bar represents mean ± SEM, n=8 per group. **p< 0.05, **p< 0.01, ***p< 0.001.*

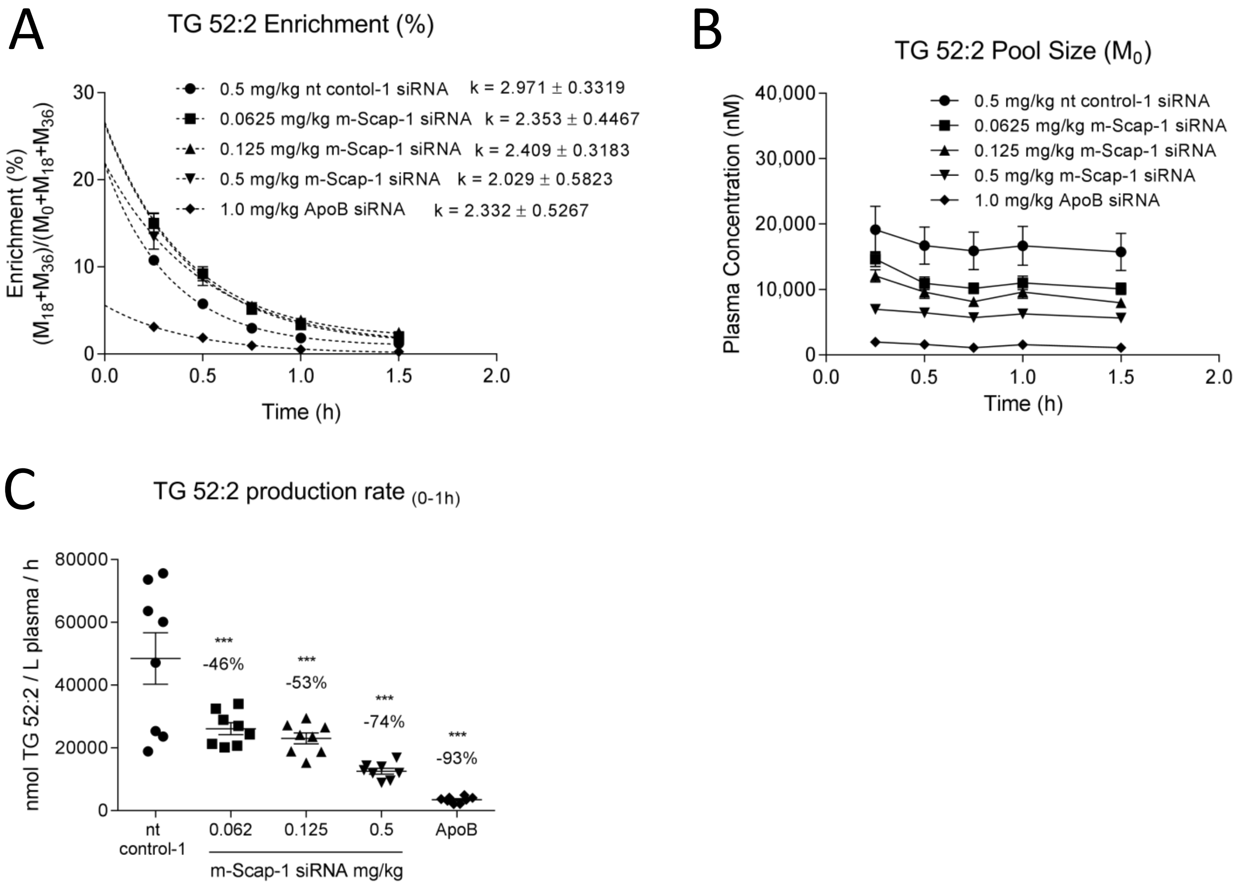

Supplemental Figure S3: Scap KD significantly reduces production of TG52:2 in C57BL/6 mice. Mice were dosed via tail vein injection with either a control siRNA at 0.5 mg/kg, or Scap siRNA at 0.0625, 0.125, and 0.5 mg/kg. One group of mice was dosed with ApoB siRNA at 1 mg/kg (IV). On day 10 after a 4 h fast, mice were administered an intravenous dose of 50 mg/kg (10mL/kg) of [$^{13}C_{18}$] oleic acid. Serial blood samples were obtained via tail nick at t=0 (pre-tracer) and 15, 30, 45, 60 and 90 minutes post-tracer administration. The concentrations of triglyceride 52:2 were determined by LC-MS/MS. (A) TG 52:2 % enrichment, (B) TG 52:2 pool size and (C) TG 52.2 production rate. Each symbol represents mean ± SEM, n=8 per group. *$p < 0.05$, **$p < 0.01$, ***$p < 0.001$*

A

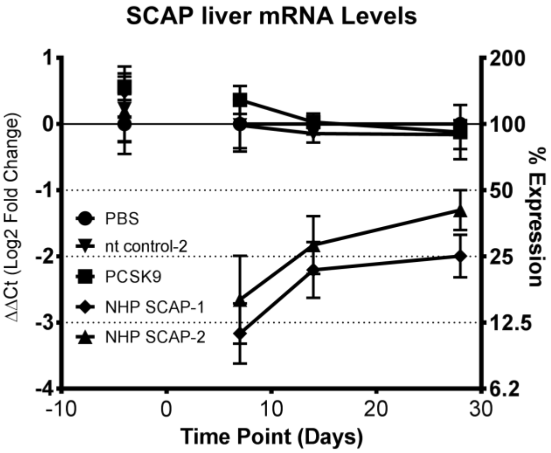

B

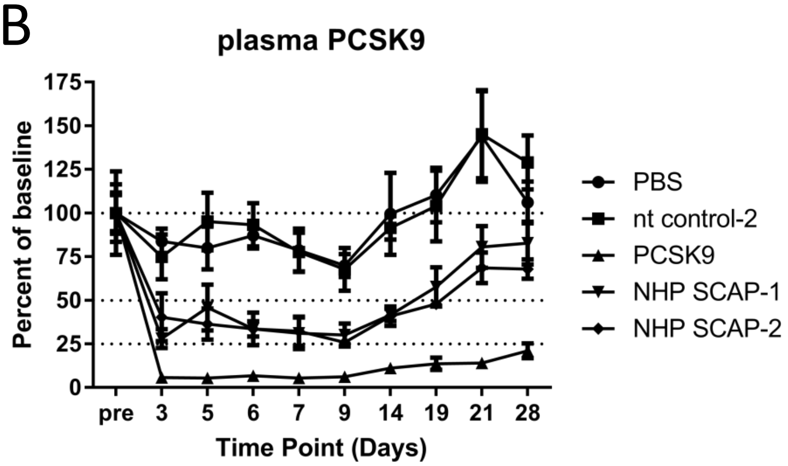

Supplemental Figure S4: siRNA sequence qualification in rhesus monkey. Lean rhesus macaque monkeys were dosed i.v. with the indicated siRNAs. Liver biopsies were collected on day -4, 7, 14, 28 and 35 after siRNA dosing for measurement of liver SCAP mRNA. Expression levels were normalized to the PBS day -4 group. (A). Blood was collected for EDTA plasma on several days as indicated in the graphs for PCSK9 analysis (B). Each symbol represents mean ± SEM, n=4 per group.

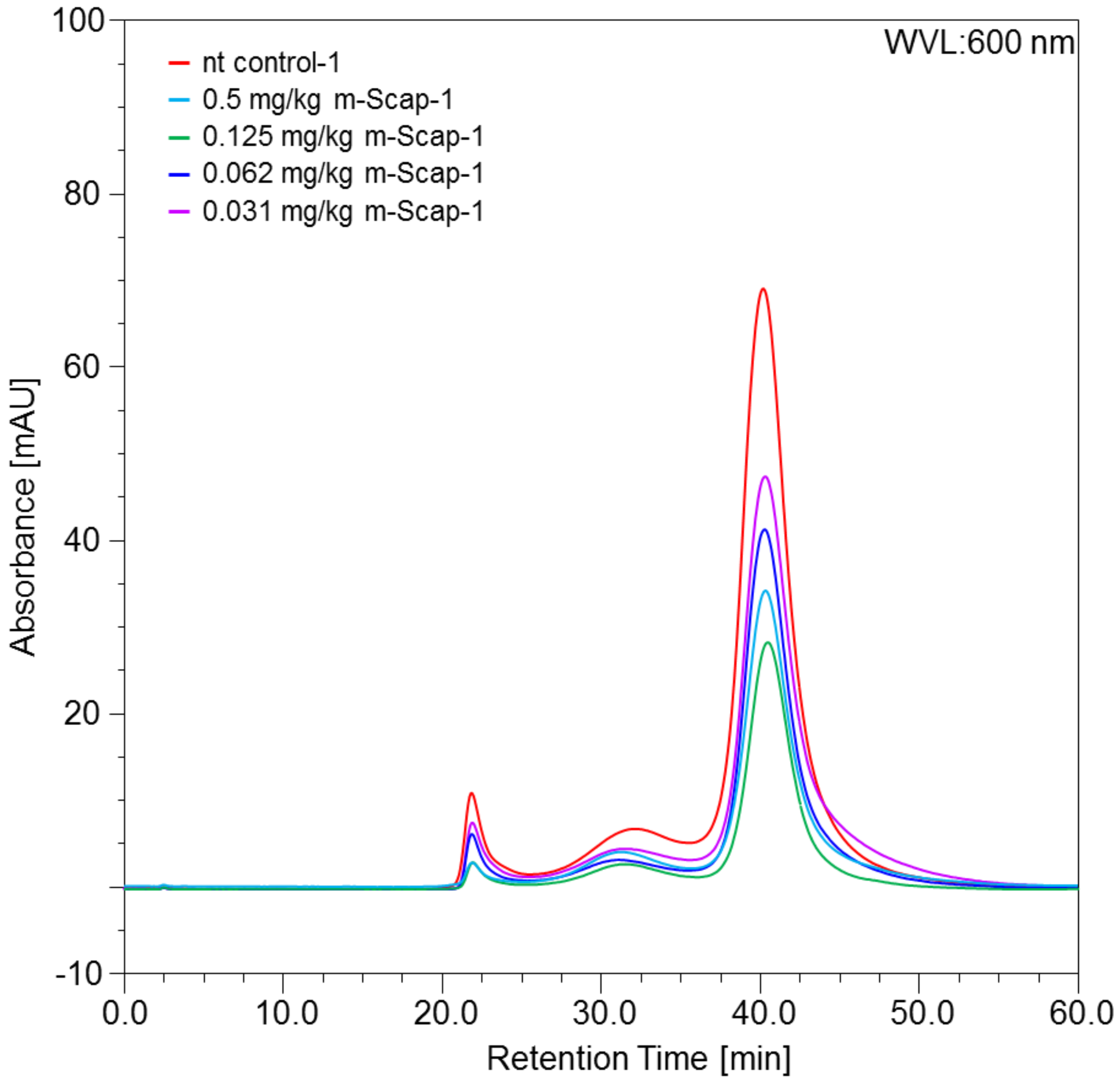

Supplemental Figure S5: Effects of Scap siRNA-LNP dose titration on plasma lipoprotein particles. Representative FPLC traces shown for each treatment group. Lipoproteins were fractionated by FPLC. The column effluent was mixed with total cholesterol E enzymatic reagent, and absorbance at 600 nm was continuously recorded. The first, second, and third peak were attributed to VLDL, LDL, and HDL, respectively.

Gene Symbol	Fold change	p-value		Gene Symbol	Fold change	p-value
ABCG1	0.56	0.003		IDI1	0.70	0.012
ABCG2	0.68	0.002		IGFBP1	0.29	0.008
ACACA	0.58	0.005		INSIG1	0.39	0.000
ACADVL	0.79	0.034		LDLR	0.59	0.023
ACSL3	0.56	0.000		LIPG	0.36	0.036
ACSS2	0.29	0.000		MBTPS1	0.84	0.017
ACSS3	0.87	0.016		NPC1L1	0.49	0.022
AGPAT4	0.69	0.032		NSDHL	0.68	0.009
AGTR1	0.79	0.032		PCCB	0.71	0.043
ALDOC	0.25	0.000		PCK1	0.62	0.000
ANGPTL3	0.41	0.000		PCSK9	0.31	0.000
APOA2	0.74	0.042		PIGQ	1.12	0.023
APOA4	0.49	0.013		PLA1A	1.34	0.040
APOA5	0.50	0.001		PPAP2A	0.77	0.017
APOE	0.77	0.004		PPARD	0.63	0.046
BAAT	0.71	0.004		PTDSS1	1.27	0.037
BID	1.16	0.048		PTPN1	0.87	0.041
CETP	0.44	0.002		RUSC1	0.47	0.001
CHKA	0.67	0.038		SCAP	0.28	0.000
CROT	1.25	0.046		SCD	0.03	0.000
CYP51A1	0.53	0.007		SIRT4	1.18	0.049
DHCR24	0.77	0.034		SLC27A2	0.74	0.014
DHCR7	0.51	0.005		SLC27A5	0.81	0.024
EBP	0.58	0.008		SLC2A4	0.21	0.005
FADS1	0.34	0.007		SPTLC1	0.90	0.016
FASN	0.32	0.019		SREBF1	0.12	0.005
FDFT1	0.70	0.011		SREBF2	0.73	0.001
G6PC	0.53	0.002		TNF	2.77	0.011
HMGCR	0.80	0.028		TPI1	1.22	0.039
HSD17B12	0.64	0.000				

Supplemental Table S1: Day 14 fold change and p-value of genes with $p<0.05$
